# Shedding Light on Hidden Methamphetamine Abuse: A Nation-wide 7-year Post-mortem Study in Taiwan

**DOI:** 10.2188/jea.JE20230263

**Published:** 2024-10-05

**Authors:** Shyh-Yuh Wei, Chien-Chou Su, Hsuan-Yun Hu, Szu-Yu Lin, Chih-Hsin Pan

**Affiliations:** 1 Department of Psychiatry, National Cheng Kung University Hospital, College of Medicine, National Cheng Kung University, Tainan, Taiwan; 2 Clinical Innovation and Research Center, National Cheng Kung University Hospital, College of Medicine, National Cheng Kung University, Tainan, Taiwan; 3 Institute of Forensic Medicine, Ministry of Justice, New Taipei City, Taiwan

**Keywords:** accidental deaths, autopsy, cause of death, multiple substances, suicide

## Abstract

**Background:**

The number of methamphetamine-related deaths has been increasing in recent decades. However, current data primarily rely on a few large-scale national surveys, highlighting the need for diverse data sources. Post-mortem studies offer advantages that compensate for the limitations of cohort studies. In this study, we aimed to (1) examine mortality rates and years of potential life lost, (2) compare proportionate mortality with previous cohort studies, and (3) quantitatively investigate causes of death as potential risk factors associated with each manner of death.

**Methods:**

We analyzed 740 cases from 2013 to 2019 in Taiwan.

**Results:**

The mean age of cases was 38.4 years, with a notable loss of 30 or more years of potential life, and 79.6% were male. The crude mortality rate was 0.45 per 100,000 person-years. The proportionate mortality indicated that autopsy dataset, compared to cohort studies, provided more accurate estimations for accidental deaths, equivalent suicides, underestimated natural deaths, and overestimated homicides. Accidental deaths were evident in 67% of cases with 80% attributed to drug intoxication. Multiple substances were detected in 61% of cases, with psychiatric medications detected in 43% of cases. Higher methamphetamine concentrations and a greater proportion of multiple substances and benzodiazepines were detected in suicidal deaths. Among accidental deaths, traffic accidents (7.9%) were the second most common cause, particularly motorcycle riders.

**Conclusion:**

Using autopsy dataset as a secondary source, we identified that over half of the cases involved drug intoxication-related accidental deaths. The significant proportion of cases involving multiple substances, psychiatric medications, and drug-impaired driving raises concerns.

## INTRODUCTION

Methamphetamine is one of the most commonly abused substances in East and Southeast Asia, and there has been a significant increase in methamphetamine-related deaths over the past few decades.^1^^–^^3^ This rising trend has resulted in worsening health and socio-economic burdens,^4^^–^^7^ as methamphetamine is responsible for a significant rate of mortality.^5^^,^^6^^,^^8^ However, most of the widely cited data were produced by a few large-scale national surveys.^9^ Moreover, those reports come from a single study design (ie, cohort study), and researchers are still confronted by many practical limitations.

One chronic problem with most prospective cohort studies is reporting or recall bias, as many measures rely on self-reported information,^2^^,^^10^^,^^11^ and the gap may be as high as 50 times.^12^ A second commonly encountered confound is the lack of biological confirmation. In some studies, even a quarter of the deaths were ill-defined or had undetermined causes,^11^ leading to underestimated results. Furthermore, both reporting bias and lack of biological confirmation may result in undetected multiple substances use. Although the stimulants-involved death rates increased independent of opioid co-involvement, multiple substances use still need to be adapted to address the evolving drug overdose epidemic.^13^

A third problem concerns many methamphetamine users with higher risk who cannot provide adequate locator information for follow-up interviews,^2^ and long-term follow-up means high drop-out rate. Unreturned participants may either cause misclassification or be excluded from the analyses in mortality consequences.^10^ Fourth, most of the studies were community-recruited and non-randomized samples, reducing the generalizability of the findings and making them unapplicable in other geographic regions.^11^

Such weaknesses (under-reported, undetermined, unreturned, and unapplicable) may inevitably be encountered and skewed by methodology-embedded inferences in cohort studies, and even those as strong as national surveys are doomed by their nature.^12^ For example, upward trends in methamphetamine overdose mortality have been observed in the past decade^2^; however, trends in suicidal or accidental deaths remains elusive. About 20 alternative methodologies exist to address hidden populations, yet they still introduce biases, such as duplicate counts, leading to unreliable estimates.^14^

Hidden drug abuse may constitute over 50% and potentially up to 90% of the total abuse.^15^ Presently, methamphetamine stands as the predominant drug of choice in this population,^16^ and this hidden aspect may be closely linked to the health burdens associated with methamphetamine use.^17^ This highlights the urgent need for a variety of data sources to better estimate the impact of methamphetamine use on national populations; otherwise, the health and social impact of methamphetamine use may be underestimated.^18^

Autopsies, which report data crucial for biological confirmation and gather last-time data from this hard-to-reach population, offer advantages that cover the disadvantages of cohort studies. Nevertheless, methamphetamine-related deaths have been a neglected area of public health research,^19^ and there is a scarcity of quantitative research on methamphetamine-related causes of death.^20^ Furthermore, certain confounding factors have not been investigated. For instance, the dataset from the justice system may potentially exhibit a higher incidence of homicides.^21^ Conversely, autopsy studies may underestimate natural deaths, since individuals who pass away in hospitals or at home with a known cause of death may not undergo autopsy examination.^22^ Therefore, autopsy studies examining the reliability and comparing them with cohort studies are warranted.

In this study, we aimed to examine the validity through mortality rates and years of potential life lost (YPLL), and further aimed to examine the reliability through proportionate mortality, in a sample of methamphetamine users who died between 2013 and 2019. We also quantitatively investigated the causes of death for potential risk factors associated with each manner of death. Given that different study designs with similar statistic methods can be used to adjust potential misleading information and provide an alternative estimation,^12^ we hypothesized that our study may enable the rendering of more accurate and reliable estimations, particularly for unnatural deaths.

## METHODS

### Details of the autopsy dataset

The Institute of Forensic Medicine, affiliated with the Ministry of Justice, serves as a national forensic institution that handles forensic autopsy cases from all regions of Taiwan, particularly for individuals who have died unexpectedly with an unknown cause of death. In Taiwan, all cases of unexpected death undergo coronial investigation, with approximately 10% of these cases subjected to autopsy by a medical examiner for cause-of-death determination (refer to eTable 1). Of the autopsied cases, 89% have been recorded in the database at the Institute of Forensic Medicine (eTable 1).

### Design, setting, and study population

We conducted a nation-wide post-mortem study to identify cases that tested positive for methamphetamine at the Institute of Forensic Medicine between 2013 and 2019. Cases were identified by searching the toxicology column in the database for the term “methamphetamine”, which included cases where methamphetamine was detected in blood samples, pleural effusion, hair, or other specimens. Methamphetamine concentrations in blood samples were also obtained from the toxicology column. A sample of the general population, matched for age and sex, was selected from the manner of death dataset in the government open data platform (https://data.gov.tw/) and from the current life expectancy tables for the population of Taiwan, published by the Ministry of the Interior of Taiwan (https://www.moi.gov.tw/cl.aspx?n=2906). The research protocol was approved by the Institutional Review Board for the Protection of Human Subjects at Antai Medical Care Corporation. This study did not include participants for whom informed consent was required.

The primary outcome was the manner of death, including accident, suicide, homicide, and natural death. The manner of death was determined by qualified forensic pathologists who performed complete autopsies, toxicology and histopathological examinations, and reviewed the available ante-mortem data and circumstantial investigations. The cause of death was coded according to the autopsy records. For cases where substances or drugs other than methamphetamine (eg, methadone, codeine, ketamine) were detected, multiple substance use was recorded as a binary indicator. However, amphetamine, the primary metabolite of methamphetamine, and ethanol, which can be produced by postmortem changes, were not included.

### Statistical analysis

The analyses were performed using R software (open source & version 4.1.0; R Foundation for Statistical Computing, Vienna, Austria), and a significance level of *P* < 0.05 (two-tailed) was used. Descriptive statistics were used to illustrate the baseline characteristics. Continuous variables were described as means with standard deviations (SDs), and categorical variables were described using numbers and proportions. Chi square tests and Kruskal-Wallis rank sum tests were used to compare the differences in characteristics and cause of death among different manners of death for categorical and continuous variables. *Post-hoc* analysis was performed using Dunn’s test for multiple comparisons of groups, and *P*-values were adjusted using the Benjamini-Hochberg method for continuous variables. For categorical variables, the Bonferroni method was used. Missing values were imputed using multivariate imputation by chained equations (MICE), and the number of participants with missing data was indicated in the footnote of each table. The method is based on fully conditional specification, where each incomplete variable is imputed using a separate model. Additional methods are reported in eMaterial 1.

#### Crude mortality

The crude mortality rate was defined as the number of deaths divided by the population (per 100,000 person-years). One potential confounder could be the proportion of autopsy and coronial investigation by year (eTable 1). We calculated the weighted mortality using inverse probability weighting (1/proportion of autopsy multiplied by the proportion of coronial investigation) for weighted estimates. To examine mortality rates over time, Poisson regression was conducted.

#### Proportionate mortality

Proportionate mortality was defined as the number of deaths assigned to a specific manner divided by the total number of deaths from all manners in the same study population. To compare the difference in proportionate mortality, the proportional difference was used. We also calculated the standard mean difference (SMD) to evaluate the effect size between methamphetamine cases and general population in a specific manner of death. The magnitude of the SMD was classified as small (0.2), medium (0.5), and large (0.8).

#### Years of potential life lost

Years of potential life lost (YPLL) is a summary measure of premature mortality. The calculation of YPLL was based on a specific manner of death, summing up deaths occurring at each age, and multiplying this by the number of remaining years to live up to a selected age limit; for example, age 75 is used in Organisation for Economic Cooperation and Development (OECD) Health Statistics. We calculated the average YPLL (AYPLL) by dividing YPLL by the number of deaths. Two-sample *t*-tests were conducted to examine between-group (methamphetamine cases and the general population) differences in AYPLL for each manner of death and sex-specific mortality.

#### Sensitivity analyses for reliability

We obtained mortality data for patients with methamphetamine use disorder from a previously published study by Lee et al (2021) and calculated the crude mortality rate, as well as the distribution of proportionate mortality. Our study covered the years from 2013 to 2019, which overlapped with the time frame of the Lee study (from 2001 to 2016), and was conducted nationwide in Taiwan, similar to the Lee study. We compared the SMD of proportionate mortality among cases in our autopsy dataset, patients with methamphetamine use disorder, and the general population, and also investigated any discrepancies in the manner of death among these three groups.

## RESULTS

A total of 740 cases containing methamphetamine were identified, and 62 cases were excluded due to an undetermined manner of death. Of the remaining cases, 454 (67%) died due to accidents, 97 (14%) due to suicide, 84 (12%) due to homicide, and 43 (6%) due to natural diseases (Table 1). Males had a higher proportion of methamphetamine cases, and this proportion was significant in cases of accidental death, suicide, and homicide (Table 1). The lowest mean age at death was observed in suicide (37; SD, 9 years), and *post-hoc* analyses indicated that the mean age at death for natural deaths was significantly higher than that for accidental, suicidal, and homicidal deaths.

**Table 1.** The baseline characteristics of methamphetamine cases

Characteristic	Natural death (N), *n* = 43	Homicide (H), *n* = 84	Suicide (S), *n* = 97	Accidental death (A), *n* = 454	*P*-value^a^	Post-hoc test^b^
Sex					0.047	male > female in H^***^, S^***^, A^***^
Male	34 (79%)	76 (90%)	72 (74%)	368 (81%)		
Female	9 (21%)	8 (9.5%)	25 (26%)	86 (19%)		
Age, years	44 (10)	39 (13)	37 (9)	39 (11)	0.002	N > H^**^, N > S^***^, N > A^*^, S < A^*^
Age group, years					0.008	
<40	14 (33%)	44 (52%)	63 (65%)	222 (49%)		
40–59	28 (65%)	36 (43%)	33 (34%)	221 (49%)		
≥60	1 (2.3%)	4 (4.8%)	1 (1.0%)	11 (2.4%)		
Methamphetamine, µg/mL	0.42 (0.52)	1.51 (4.46)	5.61 (25.09)	1.52 (4.65)	<0.001	N < H, N < S^***^, N < A^*^, S > A^**^
Dose of methamphetamine					0.012	
<0.5	32 (74%)	41 (49%)	39 (40%)	233 (51%)		
0.5–2.0	10 (23%)	29 (35%)	42 (43%)	166 (37%)		
>2.0	1 (2.3%)	14 (17%)	16 (16%)	55 (12%)		
Alcohol, g/dL	0.03 (0.05)	0.03 (0.05)	0.03 (0.04)	0.03 (0.05)	0.700	
Multiple drugs					0.002	yes > no in S^***^
No	28 (65%)	29 (35%)	32 (33%)	174 (38%)		
Yes	15 (35%)	55 (65%)	65 (67%)	280 (62%)		

The data are presented as the *n* (%) or mean (standard deviation). The missing variables were only in age (missing rate: 0.29%), methamphetamine concentration (missing rate: 20.6%), and alcohol concentration (missing rate: 21.7%). The missing values were imputed by multiple imputation by chained equations (MICE).

^a^Pearson’s Chi-squared test; Kruskal-Wallis rank sum test; Fisher’s exact test.

^b^The post-hoc test used for multiple comparisons of groups was the Dunn test, and *P*-values were adjusted using the Benjamini-Hochberg method for continuous variables. For categorical variables, the Bonferroni method was used. Adjusted *P*-value: ^*^<0.05; ^**^<0.01; ^***^<0.001.

The mean methamphetamine concentration in blood samples was 2.10 (SD, 11.29) µg/mL in this study. Furthermore, the mean methamphetamine concentration was significantly higher in suicide cases than that in accidental and natural deaths (Table 1). Multiple substances were detected in 415 (61%) cases in this sample, and this was significantly higher in suicide cases (Table 1). Cases where only methamphetamine was detected had a higher blood methamphetamine concentration (2.68; SD, 13.7 µg/mL) than cases with multiple substances (1.62; SD, 7.59 µg/mL), but this difference was not significant (*P* = 0.255).

A total of 293 (43%) cases involved the presence of psychiatric medications (eTable 2), with benzodiazepines being significantly more prevalent in suicide deaths compared to natural deaths. The most frequently detected substances, other than methamphetamine, amphetamine, or alcohol, were opioids (*n* = 195). Ketamine was present in 89 cases, while methylenedioxymethamphetamine or methylenedioxyamphetamine was present in 21 cases. Additionally, new psychoactive substances were detected in 44 cases.

### Crude mortality

**Figure 1.**
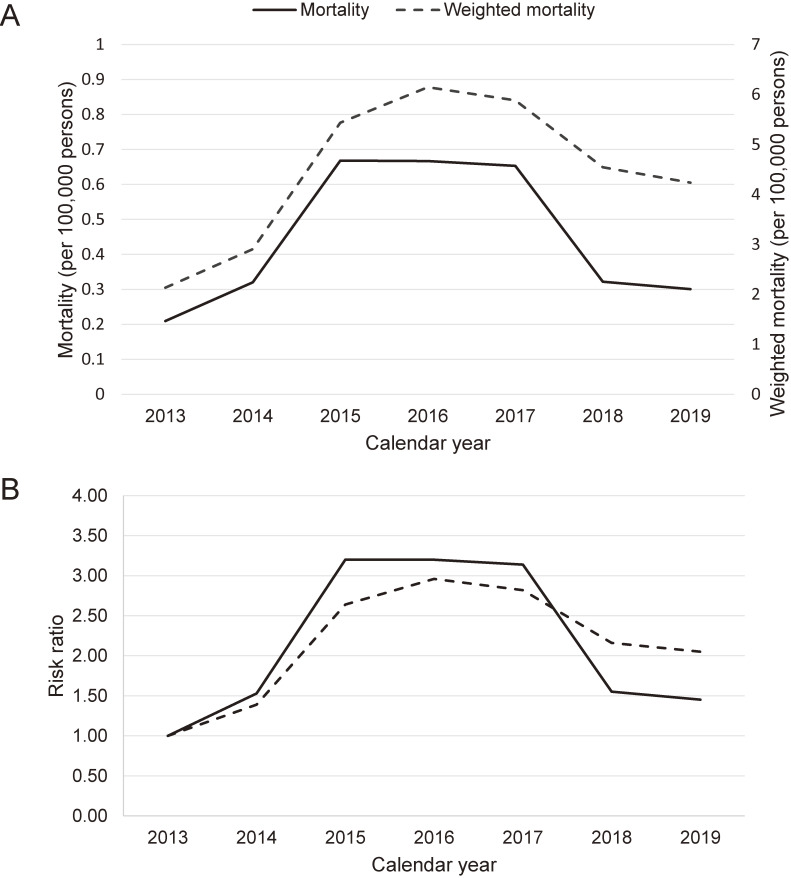
All cause methamphetamine-related population mortality rates per 100 000, by year (2013–19). (**A**) The weighted crude mortality rate increased significantly over time until 2015 (*P* < 0.001). However, the rate remained stable from 2015 to 2017 (*P* = 0.003, *P* = 0.197) and from 2018 to 2019 (*P* = 0.213). (**B**) There was a significant decrease observed during 2017 to 2018 (*P* < 0.001). Nevertheless, in 2018, the mortality rate remained 2.16 (confidence interval [CI], 1.94–2.41) times that of 2013. In summary, the methamphetamine epidemic in Taiwan appears to have reached a plateau or passed its peak.

The all-cause crude mortality rate was 0.45 per 100,000 person-years. We acknowledge that the crude mortality rate could be affected by the proportion of autopsies and coronial investigations (eTable 1). Therefore, we calculated the weighted crude mortality rate to obtain an unbiased estimate. The methamphetamine epidemic in Taiwan appears to have reached a plateau or passed its peak (Figure 1).

### Proportionate mortality

The results of proportional difference are presented in Table 2, and the SMD results are presented in eTable 2. Male methamphetamine cases of accidental death had higher proportionate mortality compared to both the general population and male methamphetamine use disorder patients. A similar pattern was found in male methamphetamine cases of homicide, which showed a small to medium effect size. Notably, male methamphetamine use disorder patients who suffered accidental death had slightly higher proportionate mortality compared to the general population, but this was not the case of homicide.

**Table 2.** The proportional difference of mortality in methamphetamine cases compared to general population

Manner of death	Sex	General population		Methamphetamine cases from autopsy		Patients with methamphetamine use disorder (Lee et al, 2021)		Proportional difference (autopsy − general population)	Proportional difference (use disorder − general population)	Proportional difference (autopsy − use disorder)

		*n*	%	*n*	%	*n*	%			
Natural death	*Total*	1,091,209	93.52	43	6.34	4,517	77.67	−87.18	−15.85	−71.33
	Male	643,828	55.18	34	5.01	3,028	52.06	−50.17	−3.12	−47.05
	Female	447,381	38.34	9	1.33	1,489	25.60	−37.01	−12.74	−24.27
Homicide	*Total*	1,158	0.10	84	12.39	22	0.38	12.29	0.28	12.01
	Male	768	0.07	76	11.21	19	0.33	11.14	0.26	10.88
	Female	390	0.03	8	1.18	3	0.05	1.15	0.02	1.13
Suicide	*Total*	26,143	2.24	97	14.31	745	12.81	12.07	10.57	1.50
	Male	17,410	1.49	72	10.62	525	9.03	9.13	7.54	1.59
	Female	8,733	0.75	25	3.69	220	3.78	2.94	3.03	−0.09
Accidental death	*Total*	48,352	4.14	454	66.96	532	9.15	62.82	5.01	57.81
	Male	34,553	2.96	368	54.28	449	7.72	51.32	4.76	46.56
	Female	13,799	1.18	86	12.68	83	1.43	11.50	0.25	11.25

In contrast, male methamphetamine cases of suicide had higher proportionate mortality compared to the general population, but no difference was observed from methamphetamine use disorder patients. It is worth noting that male methamphetamine use disorder patients who committed suicide also had higher proportionate mortality compared to the general population. For female methamphetamine cases, there were similar patterns but with smaller effect sizes as observed in male cases.

To investigate whether the results were influenced by a specific year, we divided the SMD results by year (eFigure 1). We found that male methamphetamine cases of accidental death consistently had higher proportionate mortality across all study years. Additionally, we note that there may be a bias in the estimated SMD for homicide, since the proportions were lower than 0.5%, which are considered rare events.

### Years of potential life lost

**Figure 2.**
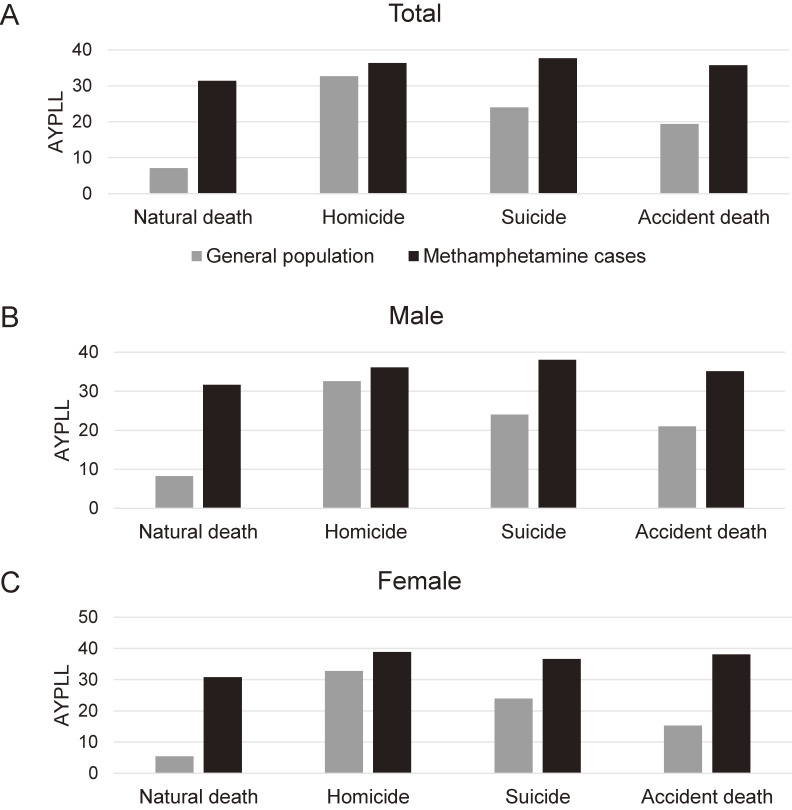
The average years of potential life lost in methamphetamine cases compared to the general population. In this sample, methamphetamine users with accidental death had an average of 35.69 (10.54) years of potential life lost (YPLL), with suicidal death 37.68 (9.32) YPLLs, and with natural death 31.44 (10.15) YPLLs. Similar results were found in both female and male groups. Notably, methamphetamine cases of natural death and accidental death had much higher YPLLs compared to the general population.

The manner- and sex-specific YPLLs are shown in Figure 2 and Table 3. Except for homicide, significant differences were observed in methamphetamine cases and the general population (*P* < 0.001).

**Table 3.** The years of potential life lost in methamphetamine cases compared to general population

Manner of death	Sex	General population			Methamphetamine cases			Mean difference^b^ (general population − methamphetamine cases) (95% CI)	*P*-value

		Deaths	AYPLL^a^	SD	Deaths	AYPLL^a^	SD		
Natural death	*Total*	*1,091,209*	*7.11*	*29.1*	*43*	*31.44*	*10.15*	*−24.3 (−27.5 to −21.2)*	*<0.001*
	Male	643,828	8.24	32.24	34	31.62	10.55	−23.4 (−27.1 to −19.7)	<0.001
	Female	447,381	5.47	23.83	9	30.78	9.02	−25.3 (−32.2 to −18.4)	<0.001
Homicide	*Total*	*1,158*	*32.7*	*21.6*	*84*	*36.38*	*13.04*	*−3.7 (−6.8 to −0.6)*	*0.0185*
	Male	768	32.58	21.48	76	36.12	13.51	−3.5 (−7.0 to −0.1)	0.0434
	Female	390	32.82	22.0	8	38.88	7.32	−6.1 (−12.3 to 0.2)	0.0574
Suicide	*Total*	*26,143*	*24.0*	*30.7*	*97*	*37.68*	*9.32*	*−13.7 (−15.6 to −11.8)*	*<0.001*
	Male	17,410	24.0	32.38	72	38.04	9.62	−14.1 (−16.4 to −11.7)	<0.001
	Female	8,733	24.0	27.0	25	36.6	8.52	−12.7 (−16.2 to −9.1)	<0.001
Accidental death	*Total*	*48,352*	*19.4*	*31.1*	*454*	*35.69*	*10.54*	*−16.3 (−17.3 to −15.3)*	*<0.001*
	Male	34,553	21.02	34.08	368	35.13	10.17	−14.1 (−15.2 to −13.0)	<0.001
	Female	13,799	15.29	22.0	86	38.1	11.75	−22.8 (−25.4 to −20.3)	<0.001

CI, confidence interval; SD, standard deviation.

^a^Average years of potential life lost (AYPLL) = years of potential life lost/number of deaths.

^b^Mean difference: the difference of mean and 95% CI were estimated by two sample *t*-test.

### Cause of death

To investigate the underlying mechanisms of each manner of death, we examined the causes of death. Among accidents and suicides, the most common underlying cause was drug intoxication (indicated as drug-related on the death certificate), accounting for 80% and 44%, respectively. Sharp force injuries were prevalent in homicides (32%), while atherosclerotic cardiovascular disease was more commonly observed in natural deaths (23%). Notably, traffic accident (7.9%) was the second most common cause in accidents, particularly among motorcycle riders (4.4%).

In cases of suicide, carbon monoxide intoxication (19%), hanging (13%), and cyanide intoxication (4.1%) were significantly more prevalent than other manners of death. Homicide cases showed a higher prevalence of assault (30%), gunshot wound (23%), rhabdomyolysis (7.1%), strangulation (4.8%), smothering (3.6%), and disseminated intravascular coagulation (2.4%) compared to other manners of death. In natural deaths, pneumonia (19%), acute myocardial infarction (9.3%), peritonitis (9.3%), myocarditis (7.0%), peptic ulcers (7.0%), acquired immunodeficiency syndrome (7.0%), cerebral aneurysm (4.7%), and meningitis (4.7%) were significantly more prevalent.

It is important to note that the presence of methamphetamine in toxicology does not necessarily imply that all cases of drug intoxication should be attributed solely to methamphetamine, particularly when multiple substances are detected. Among drug intoxication cases without multiple substances (*n* = 131), 83% of accidental deaths and 57% of suicidal deaths were attributed to methamphetamine, while 3% were attributed to alcohol, and the remainder involved multiple causes. Conversely, in drug intoxication cases with multiple substances (*n* = 416), 12% of accidental deaths and 8.3% of suicidal deaths were attributed to methamphetamine, 4.3% to alcohol, and 2.9% to opioids. Those multiple causes were significantly more prevalent in suicides compared to accidents, specifically in instances of charcoal burning, drowning, and environmental hypoxia.

## DISCUSSION

The methods used in addiction research have shaped the epidemiology of addiction and undergone two paradigm shifts: from relying on patients to relying on practitioners, and from relying on practitioners to relying on protocols in terms of prospective cohort studies.^9^ In this study, we present an alternative approach to observe the mortality of methamphetamine users using autopsy dataset, which may allow for more accurate data gathering. Accidental deaths were evident in 67% of cases (Table 1), with 80% attributed to drug intoxication, implying that more than half of methamphetamine-related deaths were due to accidents while intoxicated. Notably, previous cohort studies substantially underestimated accidental deaths (Table 2), indicating that hidden methamphetamine abusers might only come to light upon drug intoxication-related accidental deaths. The considerable prevalence of multiple substances and psychiatric medications detected (Table 1) can inform public health strategies targeting these complex drug interactions and comorbidity. Moreover, the high proportion of traffic accidental deaths, particularly among motorcycle riders, underscores the long-neglected issue of drug-impaired driving.

In the current study, the majority of cases were male and in their late 30s (Table 1), with a notable 30s YPLL (Figure 2 and Table 3), a finding consistent with longitudinal studies^10^ and an autopsy study conducted in Australia.^1^ In contrast, the all-cause crude mortality rate (0.45 per 100,000 person-years) was lower than that reported in a cohort study conducted in Taiwan (1.62 per 100,000 person-years)^3^ and an autopsy study conducted in Australia (1.03 per 100,000 person-years).^1^ The implementation of policies regarding autopsies and coronial investigations (eTable 1) can introduce additional confounding factors in autopsy studies, potentially leading to an underestimation of the crude mortality rate. Considering the limited progress in treatments, it is possible that the methamphetamine epidemic in Taiwan has reached a plateau (Figure 1). A similar trend has also been observed in the United States according to data from the Centers for Disease Control and Prevention.^23^ It is important to note that the current study presents a case series of methamphetamine-related deaths prior to the coronavirus disease 2019 pandemic. The global drug trends in recent few years have great changes, and our results may lag behind the global development. Therefore, it is possible that the levels observed after the pandemic could increase due to the immediate disruption caused by it.^24^

Although female methamphetamine cases had a lower proportionate mortality compared to males, they exhibited a significant increase in YPLL, similar to males (Figure 2 and Table 3). This indicates that although they are small in numbers, they are significantly affected by methamphetamine use. Given the significant YPLL and that four-fifths of methamphetamine users were aged 25 to 54 years,^25^ elderly methamphetamine cases were relatively scarce in our sample. However, the sample size of elderly cases was relatively higher in accidental deaths (Table 1), which is in line with previous reports on elderly traumatic patients.^26^ These two often-neglected minority groups deserve future research attention.

Our study supports the reliability of cohort studies for accessing suicides, but also highlights the hidden dangers of accidental deaths. Both methamphetamine cases of suicide and methamphetamine use disorder patients with suicide had a higher proportionate mortality compared to the general population, and there was no obvious proportional difference between methamphetamine cases and methamphetamine use disorder patients (Table 2). In contrast, while both methamphetamine cases of accidental death and methamphetamine use disorder patients with accidental deaths had a higher proportionate mortality compared to the general population, methamphetamine cases of accidental death had an even higher proportionate mortality compared to methamphetamine use disorder patients (Table 2).

The high proportionate mortality observed in accidental deaths may be attributed to the association of methamphetamine use with an increased risk of traffic accidents^27^ or more severe self-injured trauma.^28^ Among accidental deaths, traffic accidents were significantly more prevalent than other manners of death, and a substantial proportion of cases involved the detection of multiple substances, consistent with findings from a retrospective cohort study on motor vehicle accidents.^27^ Additionally, multiple substances were detected in 20% of traffic accidental deaths,^29^^,^^30^ suggesting potential drug interactions and an elevated risk of such accidents. However, as previously mentioned, cohort studies may underestimate accidental deaths due to unreturned or undetermined subjects. Another plausible explanation for this discrepancy is misclassification in terms of drug overdose.^22^ Nonetheless, in our current study, the manner of death was determined by forensic pathologists, minimizing the likelihood of misclassification.

Suicide remained a major concern in our cases, with higher methamphetamine concentration and a greater proportion of multiple substances and benzodiazepines detected (Table 1 and eTable 3). The mean methamphetamine concentration in suicides (5.61 µg/mL) was higher than the toxic plasma ranges of 5.0 µg/mL^31^ or 2.0 µg/mL^32^ in previous studies. In 67% of suicidal cases, multiple substances were detected, and our findings were supported by those from cohort studies,^2^^,^^10^ which have reported that methamphetamine users with suicidal ideation or attempt, as well as those who use multiple substances, are at a higher risk of death. Notably, the presence of psychiatric medications was noted in 43% of cases, with antidepressants being detected in 15% of suicidal cases, indicating a significant comorbidity with psychiatric disorders and a heightened risk of suicide.^33^

In comparison to cohort studies, our findings may have resulted in an underestimation of natural deaths and an overestimation of homicides. While only methamphetamine cases of homicide had a higher proportionate mortality, there was no obvious proportional difference between methamphetamine use disorder patients and the general population (Table 2). These results align with previous studies.^1^^,^^3^^,^^22^ Despite the potential overestimation of homicides, it is possible that methamphetamine users are more prone to violent mechanisms of injury, such as assault, gunshot wound, strangulation, and smothering, observed in our homicide cases. This observation is consistent with findings from previous studies conducted among trauma patients.^34^^,^^35^

Given the descriptive nature of our study, the significance testing may be interpreted cautiously. Since our autopsy dataset did not contain information on living methamphetamine users, we were unable to calculate standardized mortality ratios (SMR). Additionally, information on the duration, dosage, route, and reasons for methamphetamine use was not available and co-variables were not considered. Nevertheless, most of these data are self-reported and may contaminate the estimation. Furthermore, comorbidity, if not systematically surveyed or examined via whole-body autopsy, may mask the estimation. Thus, we believe that our approach provided a more reliable dataset, thereby enhancing the internal validity of the study by reducing the potential for biased measurements. While our results may not be generalizable to methamphetamine use disorder patients, it is worth noting that both methamphetamine and amphetamine are not prescription stimulants in Taiwan; therefore, most of the cases are related to abuse, and there is no possibility of prescription misuse. Given that nearly every person has been touched by substances in their life, those with substances in their bodies at the time of their death may reflect some similarity with substance use disorder. To address these limitations, we are currently conducting a study that links autopsy cases with Taiwan’s National Health Research Institute Database (NHIRD) for the ante-mortem data. The results will be reported elsewhere.

### Conclusion

To the best of our knowledge, this is the first quantitative autopsy study that examines its reliability and compares its findings to those of cohort studies. Our outcomes have the potential to guide public health strategies for this hidden population by providing detailed information on mortality outcome, and highlight the hidden dangers of drug intoxication-related accidental death, accounting for more than half of the cases. The identification of multiple substances in 61% of cases and psychiatric medications in 43% underscores the noteworthy comorbidity with psychiatric disorders and a heightened risk of suicide. Although the contribution of this study to understand hidden methamphetamine abuse in the international level was limited, we recognize the importance of contextualizing our findings within the broader international landscape. Our study’s design and statistical model hold applicability in other substance contexts and across different countries. As more articles of this nature are published, they can contribute to a more comprehensive understanding of hidden methamphetamine abuse at the global level.

## SUPPLEMENTARY MATERIAL

eMaterial 1. Supplementary methods and results

eTable 1. Number of methamphetamine cases seen at forensic autopsy stratified by year

eTable 2. The proportionate mortality in methamphetamine cases compared to general population

eTable 3. Number of methamphetamine cases with the presence of psychiatric medications

eTable 4. Multiple logistic regression to estimate the risk of natural death, homicide, suicide and accidental death

eFigure 1. The standardized mean difference of proportionate mortality in methamphetamine cases compared to general population by calendar years

eFigure 2. Estimated probability of suicide: age and methamphetamine dose relationship (adjusted for sex, alcohol dose, and multiple substance use)
