# Supplementary material for: Shedding Light on Hidden Methamphetamine Abuse: A Nation-wide 7-year Post-mortem Study in Taiwan
Source: J Epidemiol. 2024 Oct 5;34(10):477–84. doi: 10.2188/jea.JE20230263 (PMC11405366; doi:10.2188/jea.JE20230263)

eMaterial 1. Supplementary methods and results

Supplementary methods

Logistic regression

Multiple logistic regression was used to examine the association between demographics, methamphetamine concentration, alcohol concentration, and multiple substance use for each manner of death in methamphetamine cases. Based on the significance of the methamphetamine variable in the logistic regression model, we then plotted a scatter plot according to age, methamphetamine concentration, and probability of manner of death, with methamphetamine concentration categorized into three different concentrations: <0.5, 0.5–2.0, and >2.0, to visualize the associations.

Supplementary results

Characteristics associated with mortality

eTable 4 illustrates the association between demographics, methamphetamine concentrations, multiple substance use, and various manners of death. The estimated probability of natural death decreased with dose of methamphetamine and multiple substance use but increased with age. Furthermore, the estimated probability of homicide decreased in female after adjusted for other covariates. Even after adjusting for other covariates, methamphetamine concentration continued to increase the estimated probability of suicide (eTable 4). Stratified analysis revealed that the estimated probability of suicide increased for doses of methamphetamine over 2.0 μg/mL but decreased with older age (eFigure 2). Our findings show a notable increase in the estimated probability of suicide for methamphetamine doses over 2.0 μg/mL (eFigure 2)

eTable 1. Number of methamphetamine cases seen at forensic autopsy stratified by year

Year	Numbers of methamphetamine cases	Total amount of cases recorded in the database at Institute of Forensic Medicine	Total amount of autopsy cases in Taiwan	Total amount of coronial investigation cases in Taiwan	Weighted numbers of methamphetamine cases
2013	50	1,810	2,192	18,061	499
2014	75	2,091	2,620	18,973	681
2015	155	2,300	2,610	18,947	1,277
2016	157	2,182	2,301	20,117	1,447
2017	155	2,186	2,261	19,557	1,387
2018	77	1,361	1,478	18,941	1,072
2019	71	1,350	1,436	19,008	1,000
Total	740	13,280	14,898	148,502	7,363

eTable 2. The proportionate mortality in methamphetamine cases compared to general population

Manner of death	Sex	General population n	General population %	Methamphetamine cases from autopsy n	Methamphetamine cases from autopsy %	Patients with methamphetamine use disorder (Lee et al., 2021) n	Patients with methamphetamine use disorder (Lee et al., 2021) %	SMD[a] (autopsy - general population)	SMD[a] (use disorder - general population)	SMD[a] (autopsy - use disorder)
Natural death	*Total*	1,091,209	93.52	43	6.34	4,517	77.67	-2.91	-0.35	-1.58
	Male	643,828	55.18	34	5.01	3,028	52.06	-1.21	-0.05	-0.90
	Female	447,381	38.34	9	1.33	1,489	25.6	-1.02	-0.23	-0.55
Homicide	*Total*	1,158	0.10	84	12.39	22	0.38	0.37	0.04	0.50
	Male	768	0.07	76	11.21	19	0.33	0.35	0.04	0.47
	Female	390	0.03	8	1.18	3	0.05	0.11	0.01	0.14
Suicide	*Total*	26,143	2.24	97	14.31	745	12.81	0.33	0.30	0.04
	Male	17,410	1.49	72	10.62	525	9.03	0.29	0.25	0.04
	Female	8,733	0.75	25	3.69	220	3.78	0.15	0.15	0.00
Accidental death	*Total*	48,352	4.14	454	66.96	532	9.15	1.28	0.16	1.31
	Male	34,553	2.96	368	54.28	449	7.72	1.00	0.16	1.05
	Female	13,799	1.18	86	12.68	83	1.43	0.34	0.02	0.43

SMD, standardized mean difference.

[a]Small SMD=0.2, medium SMD=0.5, and large SMD=0.8.

eTable 3. Number of methamphetamine cases with the presence of psychiatric medications

Psychotropic drugs	Natural death n=43	Homicide n=84	Suicide n=97	Accident death n=454	P-value[a]
Antidepressants	2 (4.7%)	7 (8.3%)	15 (15%)	68 (15%)	0.11
Antipsychotics	1 (2.3%)	3 (3.6%)	5 (5.2%)	30 (6.6%)	0.7
Anticonvulsants	0 (0%)	3 (3.6%)	2 (2.1%)	5 (1.1%)	0.2
Benzodiazepines	6 (14%)	21 (25%)	37 (38%)	157 (35%)	0.011
Z-drugs	3 (7.0%)	2 (2.4%)	8 (8.2%)	13 (2.9%)	0.046
Analgesics	3 (7.0%)	2 (2.4%)	7 (7.2%)	17 (3.7%)	0.2
Others	0 (0%)	0 (0%)	1 (1.0%)	3 (0.7%)	0.8

The data are presented as the n (%). We extracted medication records from the toxicology column in the database and classified them based on their generic names. We further selected and analyzed medications that fall under the category of psychiatric medications. Polypharmacy within the same case is possible; however, medications of the same category are counted only once.

[a]Pearson's Chi-squared test; Fisher's exact test.

eTable 4. Multiple logistic regression to estimate the risk of natural death, homicide, suicide and accidental death

Variable	Natural death	Homicide	Suicide	Accidental death
	OR (95% CI)	OR (95% CI)	OR (95% CI)	OR (95% CI)
Sex (ref. = male)	1.71 (0.72–3.75)	0.38 (0.17–0.78)	1.40 (0.82–2.34)	1.03 (0.68–1.58)
Age	1.04 (1.01–1.07)	0.99 (0.97–1.01)	0.98 (0.96–1.00)	1.00 (0.99–1.02)
Methamphetamine (μg/mL)	0.41 (0.20–0.73)	0.99 (0.94–1.02)	1.03 (1.01–1.06)	0.98 (0.96–1.00)
Alcohol (g/dL)	0.25 (0.00–80.38)	4.69 (0.07–194.21)	0.20 (0.00–12.71)	1.17 (0.06–27.83)
Multiple drugs (ref. = no use)	0.24 (0.12–0.47)	1.37 (0.85–2.25)	1.33 (0.83–2.15)	1.04 (0.74–1.45)

CI, confidence interval; OR, odds ratio.

eFigure 1. The standardized mean difference of proportionate mortality in methamphetamine cases compared to general population by calendar years

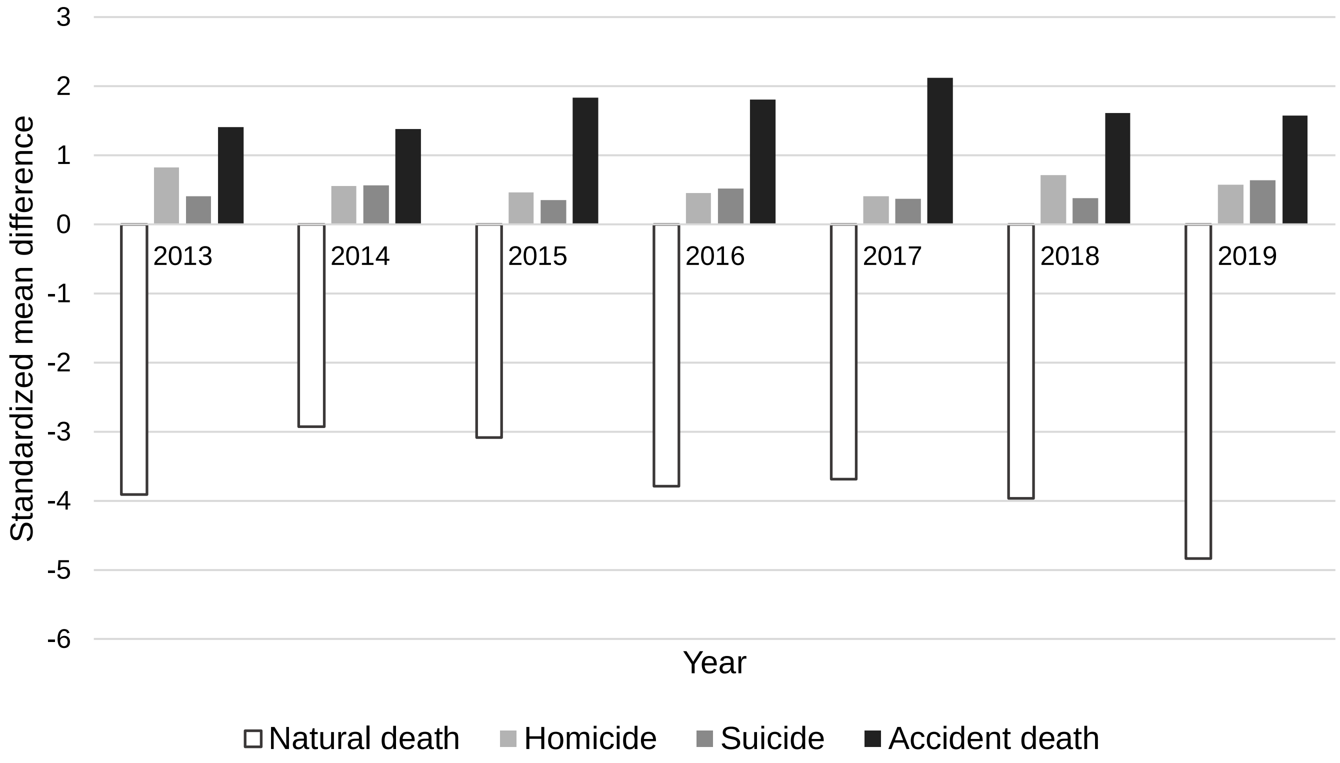

eFigure 2. Estimated probability of suicide: age and methamphetamine dose relationship (adjusted for sex, alcohol dose, and multiple substance use)

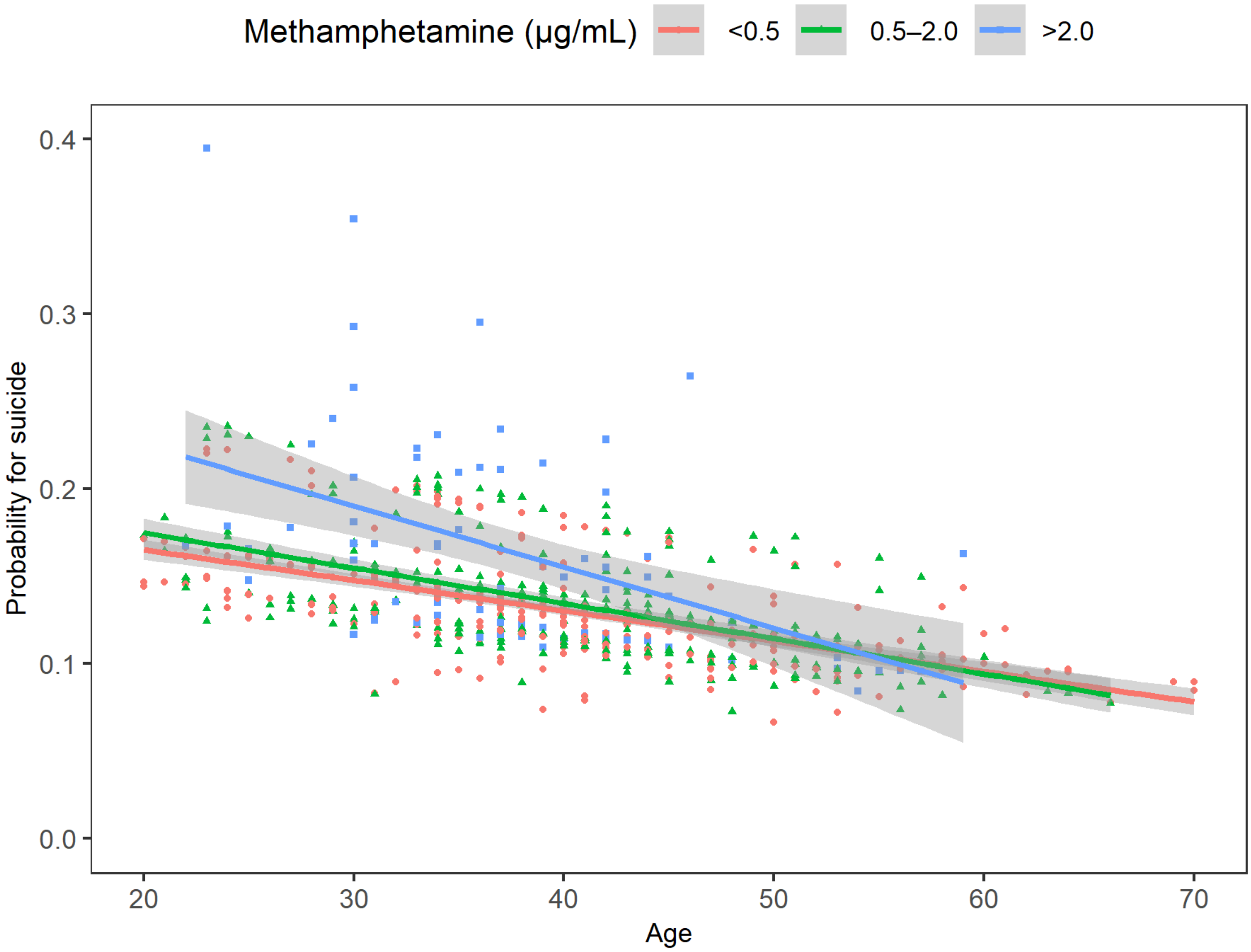

Supplement: Supplementary file 1 [file je-34-477-s001.pdf]
